# Increased Detection of Emergent Recombinant Norovirus GII.P16-GII.2 Strains in Young Adults, Hong Kong, China, 2016–2017

**DOI:** 10.3201/eid2311.170561

**Published:** 2017-11

**Authors:** Kirsty Kwok, Sandra Niendorf, Nelson Lee, Tin-Nok Hung, Lok-Yi Chan, Sonja Jacobsen, E. Anthony S. Nelson, Ting F. Leung, Raymond W.M. Lai, Paul K.S. Chan, Martin C.W. Chan

**Affiliations:** The Chinese University of Hong Kong, Hong Kong, China (K. Kwok, N. Lee, T.-N. Hung, L.-Y. Chan, E.A.S. Nelson, T.F. Leung, R.W.M. Lai, P.K.S. Chan, M.C.W. Chan);; Consultant Laboratory for Noroviruses, Robert Koch Institute, Berlin, Germany (S. Niendorf, S. Jacobsen)

**Keywords:** older children, recombinant norovirus, gastroenteritis, viruses, viral load, young adults, Hong Kong, China, GII

## Abstract

A new recombinant norovirus GII.P16-GII.2 outnumbered pandemic GII.4 as the predominant GII genotype in the winter of 2016–2017 in Hong Kong, China. Half of hospitalized case-patients were older children and adults, including 13 young adults. This emergent norovirus targets a wider age population compared with circulating pandemic GII.4 strains.

Noroviruses are leading causes of acute gastroenteritis (*1*). In the winter of 2016–2017, increased circulation of an uncommon recombinant norovirus genotype called GII.P16-GII.2 was reported in parts of Asia, including China (*2*) and Japan (*3*). Concurrently, winter norovirus cases peaked at an abnormally high level in Germany (*4*) and France (*5*) because of this emergent genotype. We report an increased detection of norovirus GII.P16-GII.2 infections in hospitalized case-patients beginning in August 2016 in Hong Kong, China. We also provide early evidence that this emergent GII.2 variant might target a wider age population than that targeted by circulating pandemic GII.4 strains.

## The Study

Since August 2012, we have conducted an ongoing molecular surveillance of norovirus genotype distribution in all hospitalized gastroenteritis patients in our teaching hospital in Hong Kong (*6*). We admitted patients on the basis of clinical severity at presentation and routinely tested for norovirus on the basis of clinical suspicion of viral gastroenteritis. We collected stool samples and tested them for norovirus by using a 1-step quantitative reverse transcription PCR assay (*6*). We then subjected norovirus RNA–positive samples to genotyping that targeted the 5′ end (region C) of the viral protein 1 (VP1) gene as previously described (*6*). After  Sanger-sequencing amplicons, we assigned norovirus genotypes by using the RIVM online norovirus genotyping tool (http://www.rivm.nl/mpf/norovirus/typingtool).

**Figure 1:**
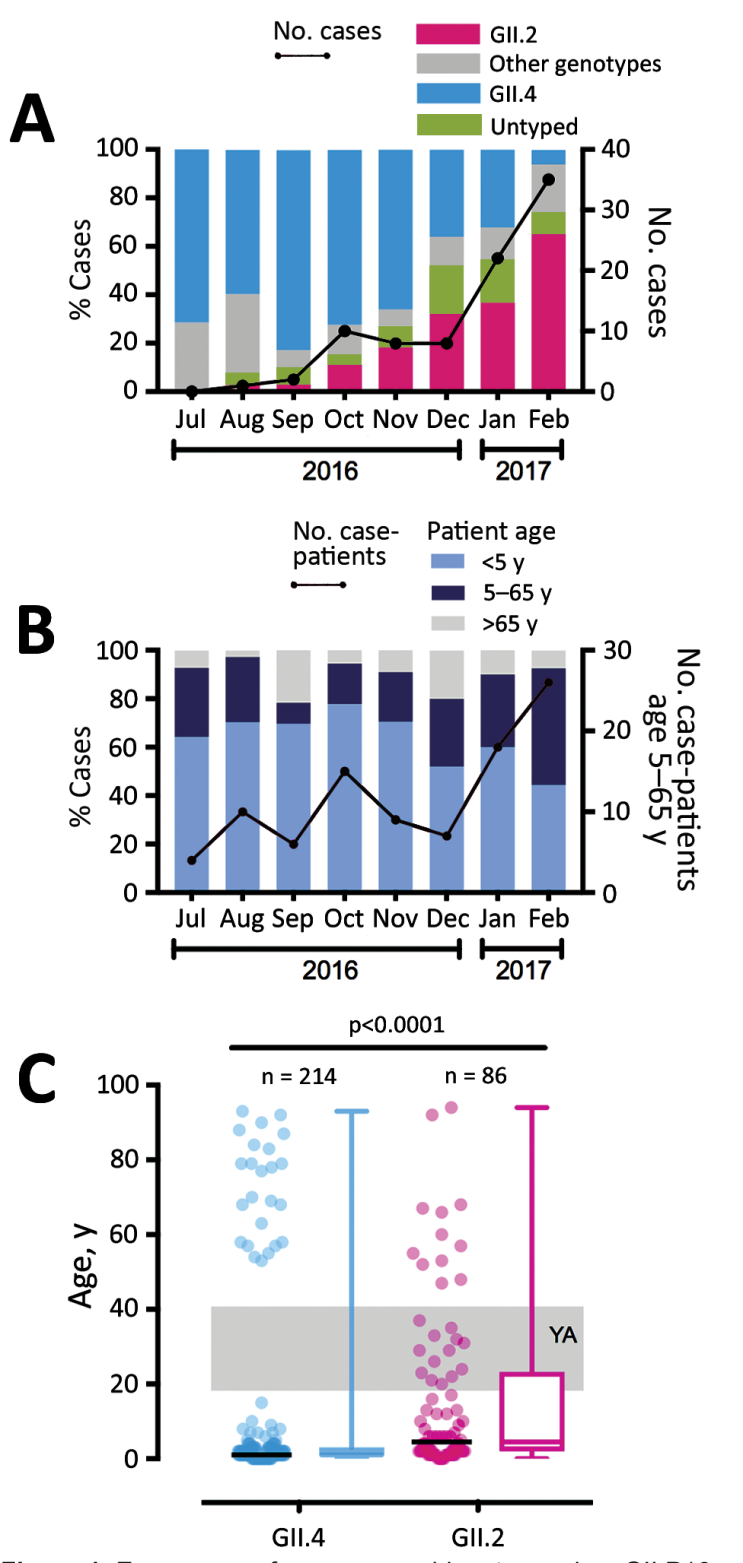
Emergence of a new recombinant norovirus GII.P16-GII.2, Hong Kong, China, winter 2016–2017. A) Distribution of norovirus genotypes, July 2016–February 2017. B) Proportion of norovirus case-patients among 3 stratified age groups. C) Age distribution of hospitalized case-patients with GII.4 and GII.2 infections. A total of 214 GII.4 and 86 GII.2 case-patients are shown. Black horizontal lines represent medians. Gray shading denotes young adults (YA; 18–40 years of age). p value calculated by using Mann-Whitney *U*-test.

During July 2016–February 2017, we collected 399 norovirus RNA–positive stool samples from 393 patients. The median patient age was 2 years (interquartile range [IQR] 1–15 years). The female-to-male ratio was 1.04:1. We successfully genotyped 357 (90.8%) samples. The top 3 circulating VP1 genotypes during the study period were GII.4 (n = 214 [54.5%]), GII.2 (n = 86 [21.9%]), and GII.3 (n = 16 [4.1%]). Before this season, GII.2 had been a rare genotype, accounting for <1% of total strains circulating locally (*6*) and <1.5% of those circulating globally (*7*). However, we observed a rapid increase in the number of GII.2 cases starting in August 2016 (Figure 1, panel A). The number and proportion of GII.2 cases increased from 1 (2.7%) in August 2016 to 35 (64.8%) in February 2017. In contrast, the percentage of GII.4 cases decreased from 71.4% in July 2016 to 5.6% in February 2017. By January 2017, GII.2 had outnumbered GII.4 as the most predominant GII genotype detected in our surveillance.

**Figure 2:**
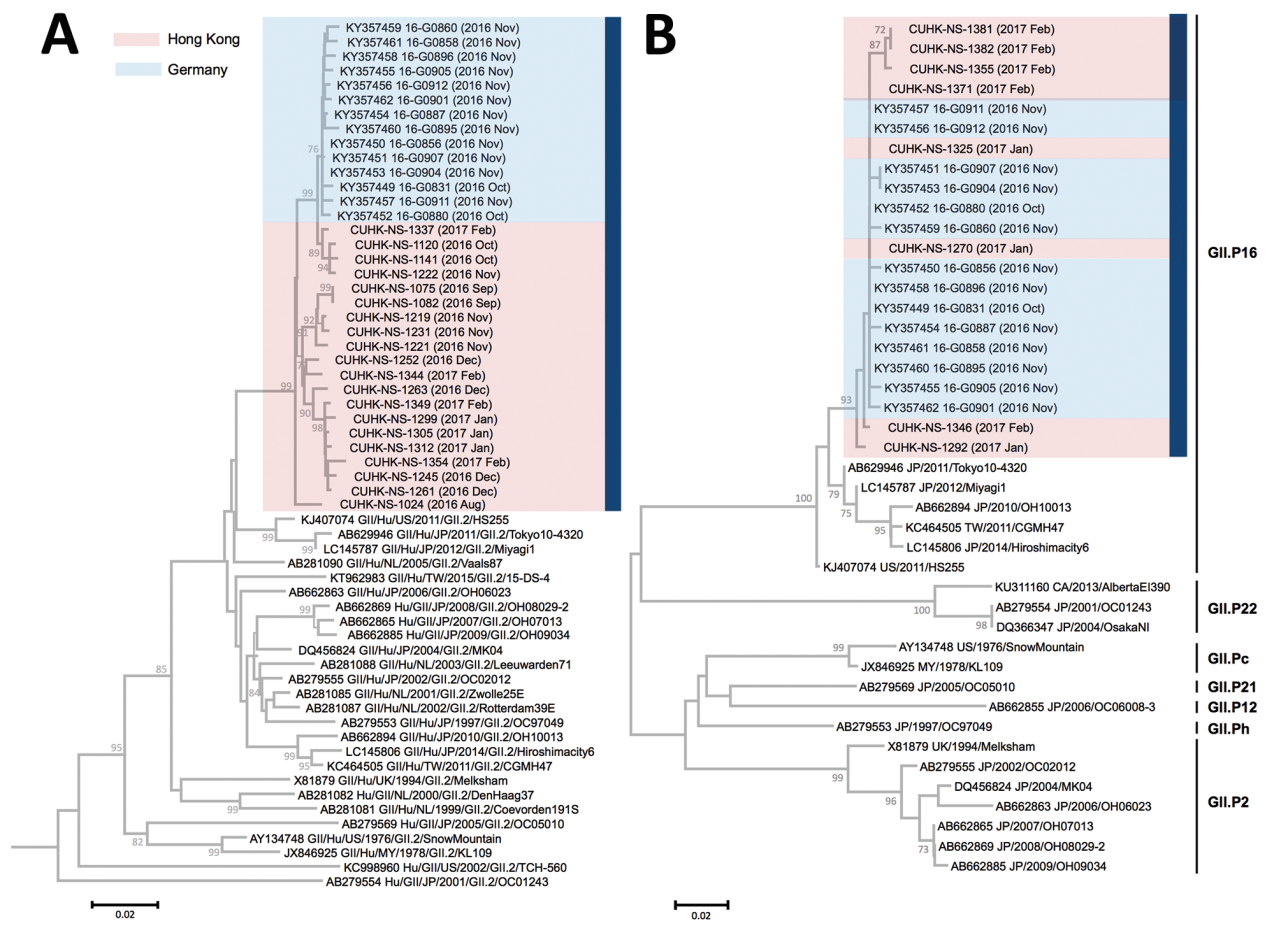
Neighbor-joining phylogenetic analysis of partial A) VP1 (1,322 nt) and B) RdRp (235 nt) gene sequences of norovirus genogroup II genotype 2 (GII.2) detected by molecular surveillance at a teaching hospital, Hong Kong, China, July 2016–February 2017. The trees were constructed by using Kimura-2-parameter distance method with 1,000 bootstrap replicates. Bootstrap values >70 (percentage) are shown at nodes. Blue bar indicates winter of 2016–2017. Pink shading denotes sequences obtained in this study. Blue shading denotes sequences from Germany during the same period. Year and month of strain collection are shown in parentheses. The VP1 tree is rooted to a genotype GII.5 strain (GII/Hu/GF/1978/GII.5/C15) (not shown), and the RdRp tree is mid-point rooted. Sequences shown in the RdRp tree were obtained from dual-typing of 8 single amplicons. All RdRp genotypes known to recombine with GII.2 VP1 are included in the RdRp tree. Scale bars are drawn to scale and indicate numbers of nucleotide substitutions per site. RdRp, RNA dependent RNA polymerase; VP1, viral protein 1.

We determined partial GII.2 VP1 gene sequences (1,322 nt in length) from the samples of 20 case-patients (GenBank accession nos. KY421044, KY677828–KY677833, and KY817742–KY817754) as previously described (*8*). We performed neighbor-joining phylogenetic inference by using MEGA 6.0 (http://www.megasoftware.net) (Figure 2, panel A). Tree topology showed that the surge of GII.2 infections in the winter of 2016–2017 in Hong Kong coincided with the emergence of a genetically distinct cluster that was different from other strains detected in Japan and Europe before 2016. Although we did not have epidemiologic data for our case-patients, formation of different subclusters in the neighbor-joining tree indicated a high genetic diversity, suggesting that emergence of GII.2 was unlikely to be an outcome of a point source outbreak. Instead, stepwise topology indicated frequent person-to-person transmission events. 

The GII.2 strains we identified clustered most closely with the recombinant GII.P16-GII.2 strains from Germany during the same period (Figure 2). Subsequent genotyping of the RNA-dependent RNA polymerase gene in 28 (33%) of the case-patients indicated that all of them were infected with strains belonging to the GII.P16 genotype. We dual-genotyped specimens collected from an additional 8 GII.2-infected case-patients during January–February 2017 by using single amplicons covering partial RNA-dependent RNA polymerase and VP1 gene regions (GenBank accession nos. KY817734–KY817741). Phylogenetic analysis confirmed that our strains belonged to the recombinant GII.P16-GII.2 variant and were not an artifact of co-infections with 2 different norovirus genotypes (Figure 2, panel B).

Epidemiologic studies have shown that norovirus GII.4 and GII.2 infections more commonly occur in young children (*9*,*10*). However, this new GII.P16-GII.2 variant might target wider age groups. We observed an increasing trend of hospitalized older children and adults (i.e., persons 5–65 years of age); 30% of all January 2017 cases and 48% of all February 2017 cases of GII.2 infection occurred in patients from this age group (Figure 1, panel B). The median age of GII.2 case-patients was significantly higher than that of GII.4 case-patients (5 years [IQR 2–23 years] vs. 1 year [IQR 1–3 years]; p<0.0001 by Mann-Whitney *U*-test) (Figure 1, panel C). The proportion of older children and adults 5–65 years of age, an age group that previously had been less commonly seen with severe norovirus infections, was significantly higher among GII.2 case-patients than among GII.4 case-patients (44% vs. 9%; p<0.0001 by Fisher exact test). More important, we observed 13 cases of GII.2 infections in young adults 18–40 years of age but no GII.4 infections in this age group (Figure 1, panel C). Among young adults 20–39 years of age, GII.2 incidence was higher than GII.4 incidence (4 vs. 0 cases/100,000 population) (Technical Appendix Figure). 

We noted a gradual narrowing in the age distribution of GII.4 infections to young children <5 years of age in the past 5 seasons (Table), presumably a result of herd immunity development, given that the current GII.4 Sydney 2012 variant has been circulating for >4 years. Part of the observed differential age distribution between GII.2 and GII.4 case-patients might be attributed to the changing epidemiology of GII.4.

**Table:** Age distribution of hospitalized patients with norovirus GII.4 infections, by season, Hong Kong, China, 2012–2017

Seasons	Median age, y (IQR)	References
2012–13	3 (1–74)	(*11*)
2012–13, 2013–14	2 (1–60)	(*6*)
2014–15	1 (1–8)	(*8*)
2015–16	2 (1–4)	Unpublished†
2016–17	1 (1–3)	This study


*IQR, interquartile range. †Chan MC. Molecular surveillance of norovirus in Hong Kong. Unpublished raw data; 2017.

## Conclusions

We report the emergence of a recombinant norovirus GII.P16-GII.2 variant that surpassed the previously predominant genotype GII.4 in hospitalized acute gastroenteritis case-patients in the winter of 2016–2017 in Hong Kong. However, unlike the recently emerged epidemic GII.17 Kawasaki variant that predominated only in part of Asia (China and Japan) during 2014–2016 (*12*,*13*), this new recombinant GII.P16-GII.2 variant also caused a steep rise in gastroenteritis cases in Asia (*2*,*3*) and Europe (*4*,*5*), indicating that it was geographically widespread across continents. We observed an increase in proportion, number, and incidence of hospitalized GII.2 case-patients in the 5–65-year age group. A similar shift in age distribution was also reported during the emergence of norovirus GII.17 Kawasaki variant in Hong Kong and Shanghai in the winter of 2014–2015 (*8*,*14*). This shift suggests a lack of preexisting herd immunity that provided a specific immunologically naive ecologic niche for this emergent norovirus to become an epidemic or even pandemic variant. A recent phylogenetic study showed that the evolution of GII.2 VP1 gene was relatively static in the past 40 years and suggested that, other than antigenicity, genetic changes in nonstructural proteins might play a role in the emergence of this recombinant GII.2 (*15*). 

Our study is limited by a small sample size, short study period, single-site setting, and lack of clinical severity evaluation. However, the infections in our hospitalized case-patients represented the severe end of the spectrum of disease, and to frequently encounter norovirus gastroenteritis in young adults in such a setting is unusual. We provide early evidence that this emergent norovirus targets an age population wider than circulating pandemic GII.4 strains do and can cause severe infections (i.e., resulting in hospitalization) apart from causing outbreaks. Collectively, these findings might have important implications for norovirus vaccine formulation and vaccination strategy. Close monitoring of the global spread of GII.P16-GII.2 is warranted.

Technical Appendix Incidence of hospitalization in case-patients with GII.4 and GII.2 infections, Hong Kong, China, July 2016–February 2017.
