# Supplementary material for: Increased Detection of Emergent Recombinant Norovirus GII.P16-GII.2 Strains in Young Adults, Hong Kong, China, 2016–2017
Source: Emerg Infect Dis. 2017 Nov;23(11):1852–5. doi: 10.3201/eid2311.170561 (PMC5652449; doi:10.3201/eid2311.170561)
Supplement: Technical Appendix — Incidence of hospitalization in case-patients with GII.4 and GII.2 infections, Hong Kong, China, July 2016–February 2017. [file 17-0561-Techapp-s1.pdf]

Article DOI: https://doi.org/10.3201/eid2311.170561

Increased Detection of Emergent Recombinant Norovirus GII.P16-GII.2 Strains in Young Adults, Hong Kong, China, 2016–2017

Technical Appendix

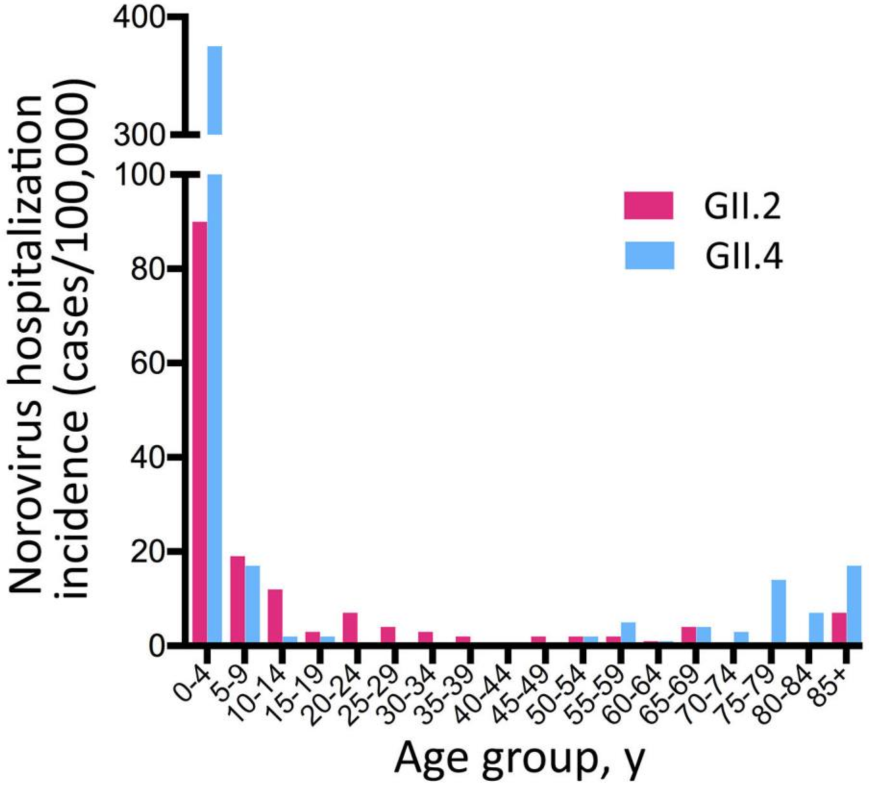

Technical Appendix Figure. Incidence of hospitalization in case-patients with GII.4 and GII.2 infections, Hong Kong, China, July 2016–February 2017. Blue bars denote GII.4 cases; magenta bars denote GII.2 cases. Incidence calculated from 2016 census data provided by the Hong Kong Census and Statistic Department.
